# Specific airway management for SVC replacement during giant anterior mediastinal tumor resection

**DOI:** 10.1186/s40981-020-00377-w

**Published:** 2020-09-14

**Authors:** Izumi Kawagoe, Daizoh Satoh, Chieko Mitaka, Masataka Fukuda, Tsukasa Kochiyama, Masakazu Hayashida

**Affiliations:** Department of Anesthesiology and Pain Medicine, Juntendo University School of Medicine, 2-1-1 Hongo, Bunkyo-ku, Tokyo, 113-8421 Japan

**Keywords:** Giant anterior mediastinal tumor resection, Airway management, Double lumen tube, Bronchial blocker

## Abstract

**Background:**

Giant anterior mediastinal tumor (GAMT) resection is a challenging procedure, for which anesthesiologist might take to need special precautions.

**Case presentation:**

A 48-year-old male patient had been scheduled to undergo GAMT resection and superior vena cava (SVC) replacement. The tumor spread surrounding SVC and left main bronchus (LMB), resulting in small volume of his left lung. A soft left-sided double lumen tube (DLT) was selected to keep the patency of LMB during left one lung ventilation (OLV) against the tumor weight. Semi-awake intubation with spontaneous breathing was selected for DLT insertion to avoid lower airway occlusion. During left OLV after right open thoracotomy, his SPO_2_ decreased below to 90%. We performed selective right upper lobe bronchial blockade using the combination of DLT and bronchial blocker. The surgery was successfully completed with this strategy.

**Conclusions:**

Although such cases are rare, they are informative for anesthesiologists, providing optional strategies.

## Background

Giant anterior mediastinal tumor (GAMT) resection is a challenging procedure [1]. Depending on the location and size of the tumor, anesthesiologists might need to take special precautions, including performing awake intubation or special respiratory support, such as percutaneous cardiopulmonary support (PCPS) [2]. We describe here a case in which we performed selective right upper lobar bronchial blockade during superior vena cava (SVC) replacement in the GAMT resection procedure, using the combination of a flexible double lumen tube (DLT) and a bronchial blocker (BB). This strategy facilitated successful completion of the scheduled surgical procedure, indicating that it can be used as an optional strategy in such cases.

## Case presentation

Written informed consent was acquired from the patient, and publication of this case report was approved by the institutional ethics committee (JHS19-011).

**Fig. 1:**
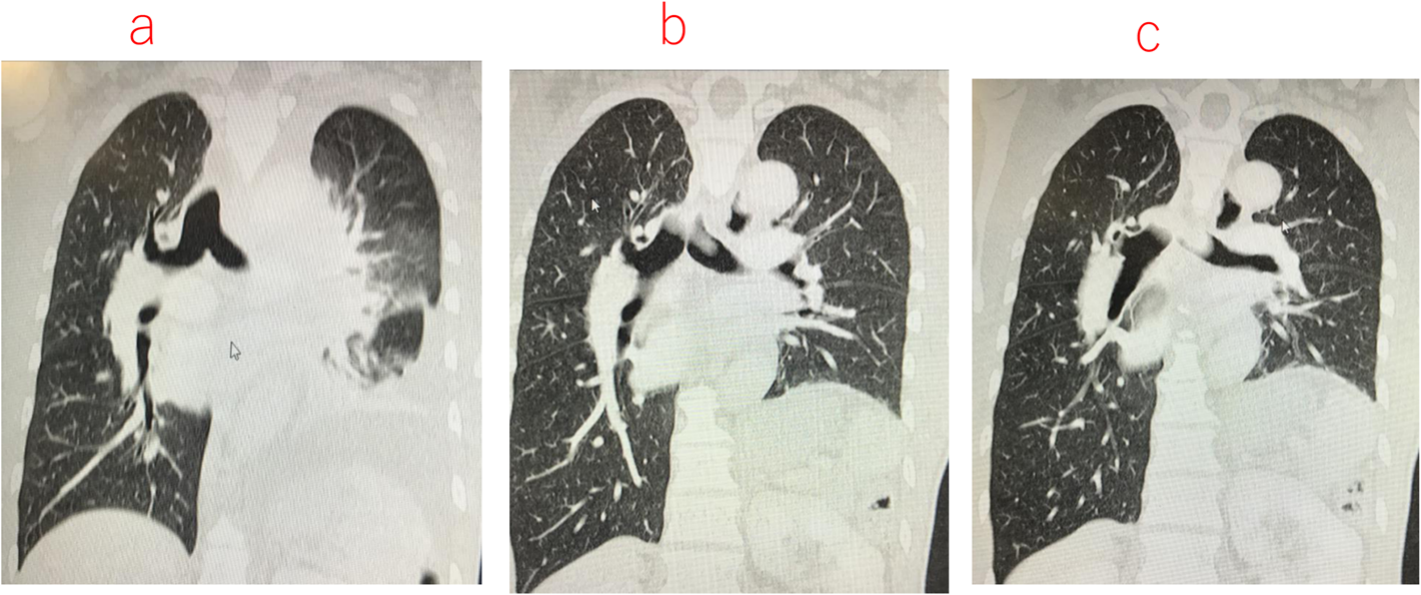
**a**–**c** CT scan showing the deformity of the left and right main bronchus. Collapsed left main bronchus due to a giant anterior mediastinal tumor (GAMT)

**Fig. 2:**
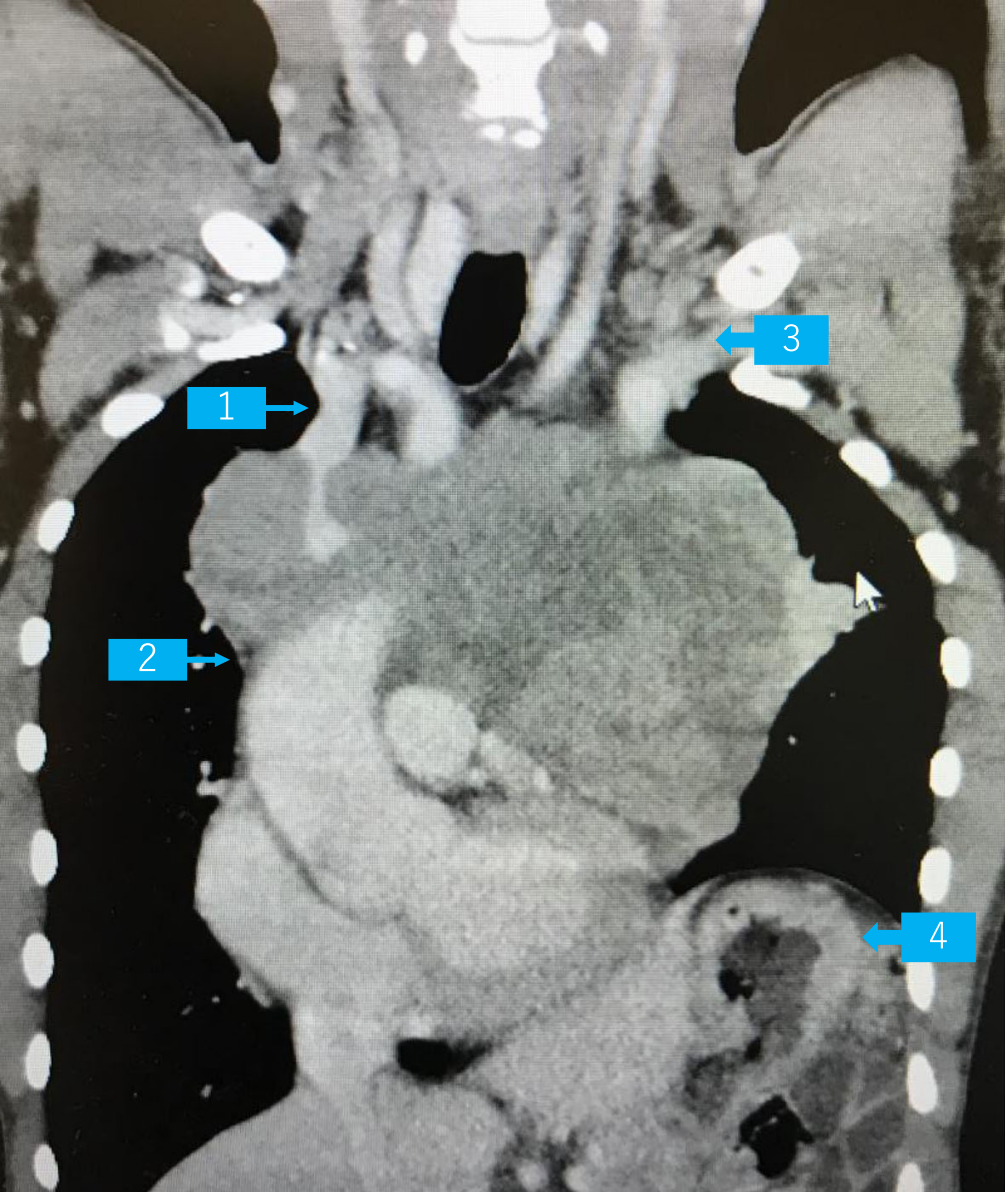
CT scan showing the location of the giant anterior mediastinal tumor. Arrow 1, right innominate vein; arrow 2, superior vena cava (SVC); arrow 3, left innominate vein; arrow 4, elevated left diaphragm

A 48-year-old male patient, 163 cm tall and weighing 53 kg, was diagnosed with atypical carcinoid tumor in the anterior mediastinum at a previous hospital and had already undergone three courses of chemo and radiation therapies. Computed tomography (CT) scans at admission (Fig. 1 a–c and Fig. 2) demonstrated that the maximal longitudinal and transverse diameters of the tumor were 11 cm and 16.5 cm, respectively. The tumor extended from the bifurcation of the innominate vein to the SVC, compressed the heart, and involved the left phrenic nerve, causing left phrenic nerve palsy and elevation of the left diaphragm. Although the left innominate vein was completely occluded, requiring preoperative heparinization, blood flow from the left jugular vein drained via well-developed collateral vessels, while flow in the right innominate vein connecting to the SVC was intact, but was surrounded by the tumor. He had ocular protrusion and remarkable dilatation of the internal jugular veins bilaterally, which was thought to be due to SVC syndrome. He had dyspnea in the left lateral position, although it decreased by changing to a forward tilt position. He could lay on his back with only slight dyspnea. The left pulmonary vein and the left upper pulmonary artery were slightly stenotic due to compression by the tumor, although blood flow in the pulmonary vessels was intact on enhanced CT images. His preoperative laboratory examinations and electrocardiogram were within normal limits. The results of respiratory function testing and arterial blood gas examination were as follows: vital capacity, 2.19 L (55.1% of normal values); FEV1.0, 1.42 L (42.5% of normal values); PaO_2_, 97 mmHg; PaCO_2_, 41.3 mmHg; BE, 0.7 mEq/L; and HCO_3_, 25.7 mEq/L.

Hence, he was scheduled to undergo surgical resection of the tumor with SVC replacement via right open thoracotomy.

## Anesthetic and surgical course

After his arrival at the operation room, routine monitors were applied and a 20-G venous cannula was inserted in his left arm. Epidural catheter was placed via the T6/7 intervertebral space in the left decubitus position. Next, after arterial catheter insertion under local anesthesia, 3 mL of 4% lidocaine was sprayed around and into his larynx and epiglottis, under oxygen administration (3 mL/min with a nasal cannula). The combination of 5 mg droperidol and 100 μg fentanyl was intravenously administered to induce light sedation. A 37-Fr left-sided silicon DLT with a reinforced bronchial tip (SILBRONCHO™, Fuji Systems, Tokyo, Japan) was inserted via his trachea into the left main bronchus using a bronchoscope with 3.8 mm diameter (Ambu aView™ and aScope™ 3 Broncho Slim, Ambu, Copenhagen, Denmark), with the patient breathing spontaneously. Although remarkable stenosis of the left main bronchus (LMB) was seen under fiberoptic bronchoscopy (FOB), the bronchoscope was passed successfully, serving as a guide for placement of the DLT into the LMB. After bronchial insertion of the DLT and confirmation of the adequacy of manual positive pressure ventilation, 100 mg of propofol and 50 mg of rocuronium were administered for anesthesia maintenance. Despite collapse of the anterior and left apical parts of the bronchus by the weight of the tumor, accurate placement of the left-sided DLT could be performed owing to its flexibility. During left-sided one lung ventilation (OLV), positive pressure ventilation could be achieved with a permissive tidal volume of 400 mL after muscle relaxant administration in the supine position. Then, a central venous catheter was inserted via the right femoral vein.

After the patient was turned to the left lateral position, we once again confirmed the accuracy of DLT placement using bronchoscopy. At this point, his systolic blood pressure decreased to 60 mmHg, probably due to shift in the SVC that resulted in reduced venous return. Intermittently, 0.1-mg boluses of phenylephrine were administered as required, up to a total of 0.5 mg. However, since this did not result in an adequate increase in blood pressure, a continuous infusion of phenylephrine at the rate of 1 mg/h and albumin administration for volume loading were commenced. After achieving circulatory stability, left-sided OLV was attempted for 30 min. Since SpO_2_ could be maintained above 93% (FiO_2_ = 1), we decided to start the scheduled surgical procedure.

**Fig. 3:**
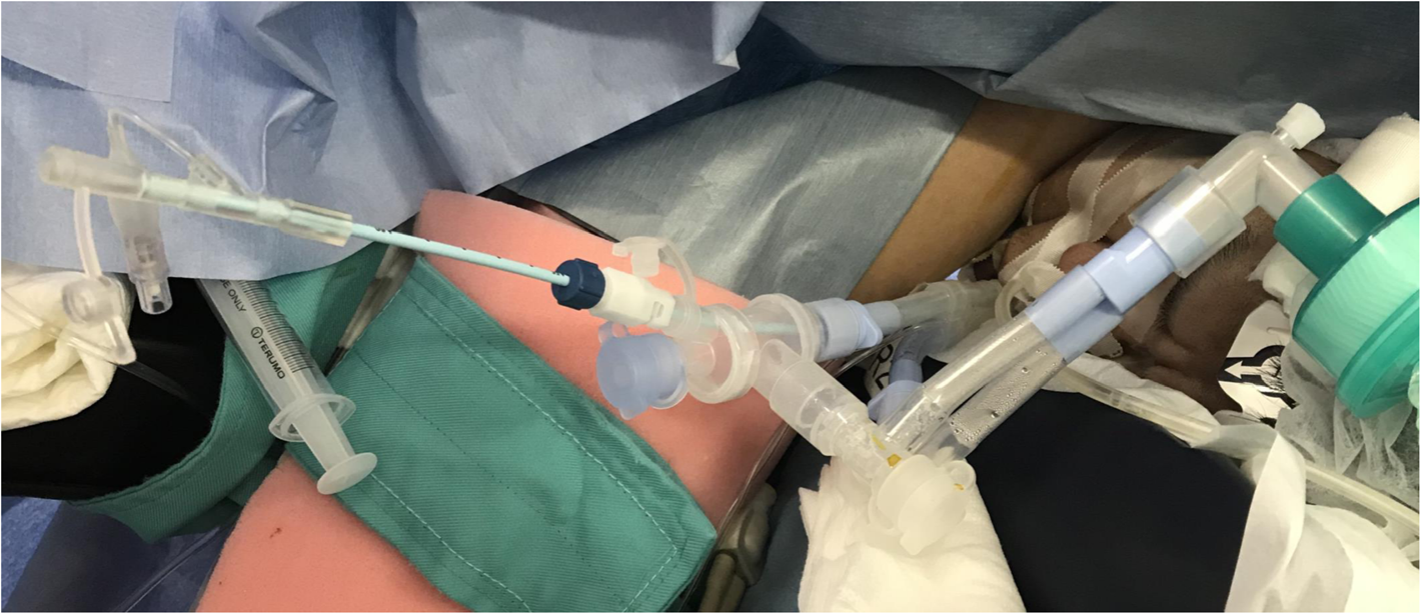
Intraoperative use of the left-sided double lumen tube and bronchial blocker. The bronchial blocker (BB) was inserted through the tracheal lumen of the left-sided double lumen tube (DLT). The left lung was ventilated through the bronchial lumen of the DLT, and the right middle and lower lobes were ventilated through the tracheal lumen of the DLT. The right upper lobe was partially blocked with the BB

**Fig. 4:**
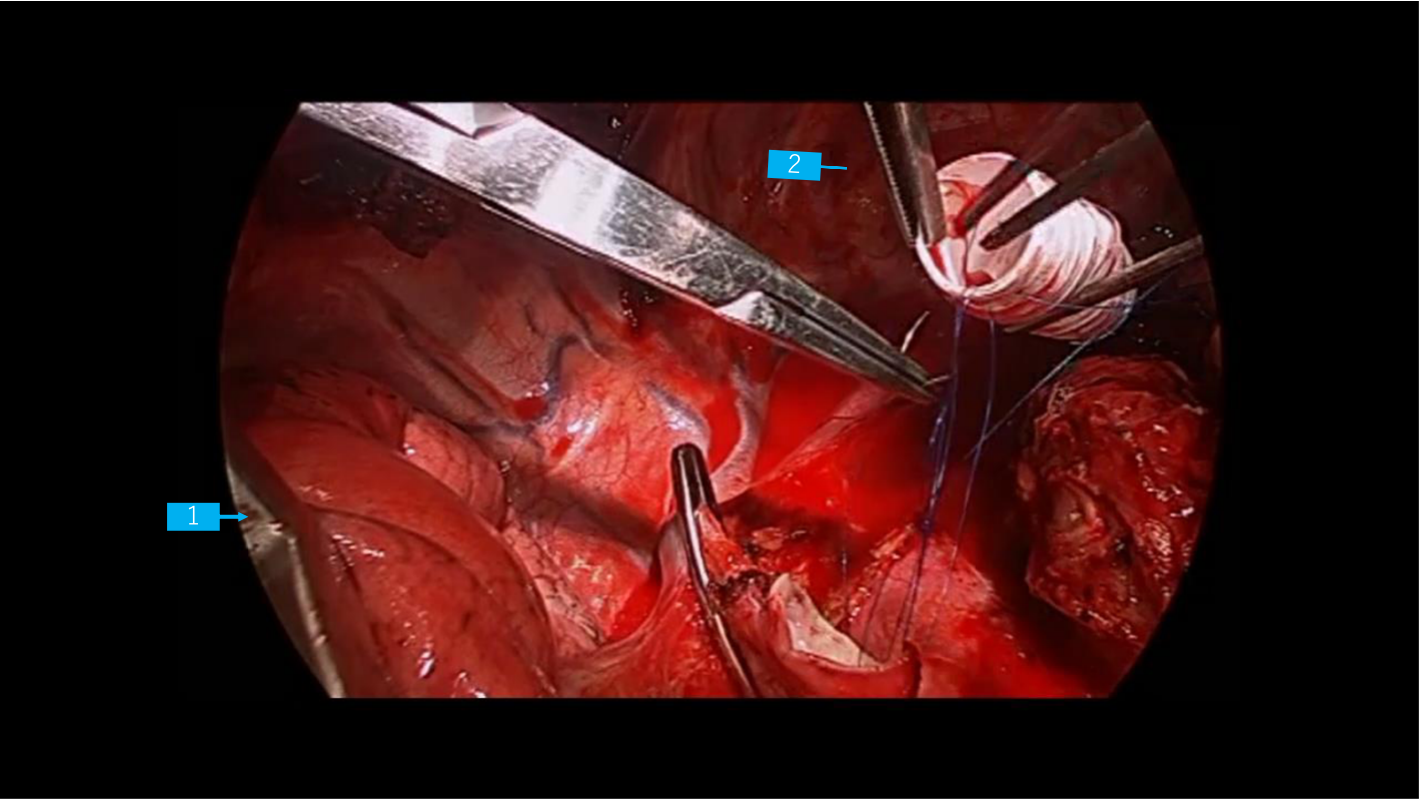
Intraoperative thoracoscopic view. The main procedure of superior vena cava (SVC) replacement that was performed during selective blockade of the right upper lung lobe using a bronchial blocker. Arrow 1, collapsed right upper lobe; arrow 2, artificial vessel that was used to replace the superior vena cava

However, on opening the right thoracic cavity, his SpO_2_ gradually decreased to 88%, although the FiO_2_ remained at 1.0. Hence, we considered alternative airway management strategies or changing the surgical procedure before SVC clamping, because we have previously experienced severe hypotension and hypoxemia during SVC dissection that was difficult to manage. It was clarified by direct observation via the surgical field that selective block of the right upper lobe was the only available option during the main procedure of SVC replacement. Hence, we decided to perform selective block of the right upper lobe with a BB (Uni-blocker™ Fuji Systems Corp, Tokyo, Japan) in combination with the left-sided DLT (Fig. 3). The BB was inserted first through the tracheal lumen, followed by a smaller (3.1 mm) diameter FOB (LF-DP ™, OLYMPUS, Tokyo, Japan). BB was easily inserted with a simple rotation technique into the right upper bronchus, appropriate placement of BB in the upper bronchus being facilitated by the slight stenosis of the intermedius just below the right upper bronchus (Fig. 1a, b). Thereafter, SpO_2_ could be maintained above 93% during the remaining procedure and SVC replacement was successfully performed (Fig. 4).

The tumor along with the SVC and part of the sternum and the right fourth rib were resected. Additionally, after turning the patient to the supine position, partial resection of the right lung and left upper lobectomy were performed due to tumor invasion. After tumor resection, there was no decrease in either blood pressure or SpO_2_. Postoperatively, the DLT was changed to a supraglottic airway device (SGA) to facilitate subsequent bronchoscopic observation and to avoid excessive airway pressure due to bucking or coughing during emergence from general anesthesia.

Subsequently, the patient’s spontaneous respiration recovered after reversal of the muscle relaxant with 200 mg sugammadex, and the SGA was removed easily and smoothly. Durations of surgery and anesthesia were 443 and 525 min, respectively. Postoperative recovery was favorable. He was discharged without any complications on the tenth day after surgery.

## Discussion

In our case, a case conference between surgeons and anesthesiologists was held 10 days preoperatively, and the potential problems and strategies required to avoid and/or cope with them were discussed and planned.

Remarkable deformity of the left and right main bronchus might impair placement of the standard left-sided DLT. In such cases, alternative airway devices need to be selected. We first considered the possibility of right-sided DLT insertion, since there was less deformity of the right main bronchus as compared to the left. However, since we knew that left OLV would be required intraoperatively, and that this was likely to be a problem due to the LMB being surrounded by the tumor and hence would potentially be occluded due to the effect of gravity after turning the patient to the left lateral position, leading to possible ventilatory failure, we decided to insert the DLT on the left side to maintain patency of the LMB. Consequently, we selected a specific flexible left-sided DLT that has a reinforced bronchial lumen made of soft silicon, allowing intraoperative manipulation of the bronchus while still preventing its collapse.

We anticipated development of lower airway obstruction after general anesthesia induction, due to the weight of the GAMT compressing the carina and main bronchus. This obstruction could be overcome using the specific left-sided DLT. Moreover, DLT insertion with spontaneous breathing was planned to avoid a “cannot manually ventilate” situation after general anesthesia induction, especially when spontaneous breathing was stopped.

Due to prior injury to the left phrenic nerve, the diaphragm was shifted upward and the left lung volume was remarkably decreased, suggesting the possibility of insufficient oxygenation during left-sided OLV and right open thoracotomy. Although we discussed the possibility of other surgical approaches, such as left open thoracotomy or sternotomy, with the surgeons, they determined that they would not be able to reach the right innominate vein and SVC via any other approach due to severe adhesions between the tumor and sternum.

In view of the possibility of the worst-case situation of hypoxemia during OLV, we had to prepare additional devices. Veno-venous extracorporeal membrane oxygenation (VV-ECMO) via his femoral vein [3], a continuous positive airway pressure (CPAP) system, and BB for selective lobar blockade were prepared. In addition to these, at the beginning of left OLV, we had planned to provide a more than 30 min trial period of OLV before SVC clamping. In a previous report, serious hypoxemia during OLV was treated with high-frequency jet ventilation [4, 5].

The airway management of GAMT can be life-threatening. Particularly in case the tumor compresses the lower airway, it is important to be well-prepared for all potential adverse scenarios, and to discuss and plan strategies and procedures by cooperation between surgeons and anesthesiologists. At the same time, we need to be aware about alternative airway devices that can enable performance of innovative management strategies.

## Data Availability

Not applicable.

## Abbreviations

GAMT: Giant anterior mediastinal tumor
SVC: Superior vena cava
DLT: Double lumen tube
PCPS: Percutaneous cardiopulmonary support
OLV: One lung ventilation
LMB: Left main bronchus
FOB: Fiberoptic bronchoscopy
BB: Bronchial blocker
SGA: Supraglottic airway device
CPAP: Continuous positive airway pressure
HFJV: High-frequency jet ventilation
